# Implementing Digital Storytelling for Health-Related Outcomes in Older Adults: Protocol for a Systematic Review

**DOI:** 10.2196/15512

**Published:** 2019-12-20

**Authors:** Jennifer Stargatt, Sunil Bhar, Jahar Bhowmik, Abdullah Al Mahmud

**Affiliations:** 1 Faculty of Health, Arts and Design Swinburne University of Technology Hawthorn Australia

**Keywords:** aging, health, psychology, mental health, memory

## Abstract

**Background:**

The number of older adults is increasing rapidly worldwide. Older adults face a unique set of challenges and may experience a range of psychological comorbidities. Advances in multimedia technology have allowed for digital storytelling to be utilized as an intervention for health-related outcomes.

**Objective:**

The primary aim of the proposed systematic review is to examine the reported health-related outcomes for older adults engaged in digital storytelling. The review also aims to examine the methods associated with digital storytelling, characteristics of digital story products, and implementational considerations.

**Methods:**

This protocol adheres to the recommendations of the Preferred Reporting Items for Systematic Reviews and Meta-Analysis Protocols. We will systematically search selected electronic databases to identify studies that meet our eligibility criteria. From the included studies, data will be extracted and synthesized using a narrative approach and summarized in tables. The methodological quality of the included studies will be assessed using the Mixed Methods Appraisal Tool.

**Results:**

Systematic searches, data extraction and analysis, and writing of the systematic review are expected to be completed by the end of 2019.

**Conclusions:**

The proposed systematic review will summarize the existing studies using digital storytelling to improve health-related outcomes for older adults. Results from this review will provide an evidence base for the development of digital storytelling interventions that are effective and implementable with older adults.

**International Registered Report Identifier (IRRID):**

PRR1-10.2196/15512

## Introduction

### Background

By the year 2050, it is anticipated that the number of people worldwide aged 60 years and older will double to 2.1 billion, and the number of people aged 80 years and older is expected to triple [1]. Although the majority of older adults enjoy health and happiness in their later years, a unique set of challenges are faced by this section of the community—for example, many will experience the loss of family and friends, a decline in physical and cognitive ability, and an increased dependency on others and many will undergo uncertainty as they anticipate impending death. As a result, many older adults experience poor mental health and live with several psychological comorbidities including depression and anxiety [2], social isolation, and loneliness [3,4]. Those with depression and anxiety report poorer quality of life than those with just comorbid physical conditions [5], and the presence of poor physical health, along with psychological conditions, stressful life events, and a lack of social connectedness, has been linked to suicide in late life [6]. These compounding difficulties highlight the importance of developing innovative interventions for improving and maintaining psychological health in later life.

Storytelling in later life through reminiscence has been recognized as an activity to support well-being in later life [7]. Recalling past events, in reminiscence, life review and other approaches may improve psychological well-being through several mechanisms. Such mechanisms may include encouraging a positive sense of self, eliciting pleasant memories to reduce negative mood states, promoting beliefs of self-mastery and ability to problem solve, and supporting ego integrity—the ability to accept one’s highs and lows and to integrate past experiences and find a meaning or greater purpose in the events of one’s life [8]. In telling their story, older adults may benefit from the opportunity for emotional expression, the ability to express their identity, and the experience of being listened to [9]. Reminiscence and life story work with older adults has often produced tangible artifacts such as storybooks, collages, and memory boxes [10], so that stories can be recorded, kept, and shared with others.

Owing to the advances in the capability and accessibility of multimedia technologies, it is now possible to produce digital life story artifacts with relative ease. Digital storytelling is a process that involves using multimedia technology to combine images, sounds, and narration into a film that documents one’s lived experiences [11]. It has been used across disciplines in a variety of ways, largely utilized in educational settings [12,13], participatory research [14,15], and community engagement [16]. It can be facilitated with groups or with individuals, with a view to engaging participants in recording and sharing their lived experiences to educate others [17], to preserve stories and strengthen community bonds [18], and to assist participants in deepening their understanding of their lives and circumstances [19].

The use of digital storytelling to improve the health of older adults is an emerging area of research, with studies employing significantly varied methods published across a range of disciplines. Such studies suggest that digital storytelling may be used with older adults as a tool to improve mood [20,21], enhance memory [21], increase social connectedness [20,22,23], encourage personalized care practices among those who require it [23], and promote intergenerational learning [24,25].

To date, no published systematic review has evaluated the methods for and health-related outcomes of digital storytelling for older adults. This protocol proposes a review that aims to systematically examine the current state of digital storytelling with older adults, concerning outcomes of digital storytelling, methods for digital storytelling, and characteristics and content of the resulting digital story products. It also aims to examine factors related to the implementation of digital storytelling—this may include outcomes such as acceptability, feasibility, adoption, and adherence [26]. It aims to do so to provide a model for future digital storytelling interventions that promise to be effective and implementable for their intended purpose.

### Objectives

This systematic review aims to answer the following questions:

1. What are the health-related outcomes of digital storytelling with older adults?
2. How does the process of, and the products created by, digital storytelling vary?
3. What are the implementational considerations in digital storytelling with older adults?

## Methods

### Protocol and Registration

This protocol was developed in adherence with the recommendations of the Preferred Reporting Items for Systematic Review and Meta-Analysis Protocols guidelines [27]. The systematic review will also adhere to the recommendations of the Preferred Reporting Items for Systematic Reviews and Meta-Analyses (PRISMA) guidelines [28]. This protocol is under review for registration with the International Prospective Register of Systematic Reviews.

### Eligibility Criteria

#### Study Designs

Given that digital storytelling remains a relatively new area of research across various health care disciplines, it is anticipated that there is considerable heterogeneity in study designs. Relevant research has taken the form of quantitative, qualitative, and mixed methods studies. We will be inclusive in our eligibility criteria: we will review a range of study designs, including quantitative (eg, randomized, nonrandomized, quasi-randomized, and cluster randomized controlled trials; pilot trials; open trials; case studies; cross-section studies; cohort studies; and case-control studies), qualitative, and mixed methods studies, provided that at least one health-related outcome is reported in relation to digital storytelling participation (see the Outcomes section below).

#### Participants

We will include studies in which participants are older adults, defined for the purpose of this review according to the United Nations classification as those aged 60 years or above [1]. We will exclude studies if the age range for the sample begins under 60 years, even if the mean age is above 60 years; studies will only be included if all participants are identified as being aged above 60 years. Participants will not be excluded on the basis of their health—samples of participants may include those with dementia or mild cognitive impairment and other age-related illnesses. Participants may reside in the community or institutional settings, such as long-term care facilities, retirement communities, hospices, palliative care, and hospitals.

#### Intervention

Interventions of interest will be those that engage participants in digital storytelling. We will define a digital story as a short (eg, 3-5 min) multimedia clip (eg, images, videos, narration, and music) that is centered on the lived experience of older adult participants. There may be varying levels of participant involvement in technical production; for example, digital stories may be produced entirely by participants or produced on their behalf partly or wholly by others such as researchers, carers, or volunteers.

Studies will be included if their primary aim was to assess digital storytelling outcomes for participants other than older adults (eg, participants engaged in creating digital stories with older adults to assist with their understanding of the challenges of old age) and if health-related outcomes for older adults are reported as additional outcomes. Studies will be excluded if digital storytelling was used in conjunction with another intervention where the effects of digital storytelling alone are not reported or cannot be ascertained.

#### Comparator Groups

Any type of control group may be present in studies included for review.

#### Outcomes

This systematic review is primarily interested in the health-related outcomes for older adults who participate in digital storytelling. Such health-related outcomes may include mood, memory, quality of life, and social engagement. These outcomes may be measured and reported quantitatively (eg, using validated psychometric assessment tools) or qualitatively (eg, as a result of participant interviews that were transcribed and analyzed thematically). In addition, secondary outcomes include (1) process characteristics (ie, information related to the process of digital storytelling, eg, length of participation and level of involvement in technical production), (2) product characteristics (ie, variation in digital stories produced as a result of a digital story process, eg, length of story and presence of audio-visual components such as still photographs, videos, music, and narration), and (3) factors related to the implementation of digital storytelling with older adults—this may include acceptability, adoption, appropriateness, feasibility, fidelity, cost, coverage, and sustainability outcomes [26].

#### Report Characteristics

We will include studies for which we can access the full-text reports, which were published in scholarly journals or unpublished in the case of dissertations and theses and were written in English with no restrictions on the country of origin or the year of publication.

### Search Methods for Identification of Eligible Studies

We will conduct a search of the following databases using a planned search strategy to identify published studies: MEDLINE (Scopus), Embase (Scopus), PubMed, PsycINFO, Web of Science, Cumulative Index of Nursing and Allied Health Literature (EBSCO), Academic Search Complete (EBSCO), Abstracts in Social Gerontology (EBSCO), Psychology and Behavioral Sciences Collection (EBSCO), Health Source: Nursing/Academic Edition (EBSCO), and SocINDEX (EBSCO). Unpublished studies will be identified by searching ProQuest Dissertations and Theses and Open Access Theses and Dissertations.

Selected search terms were chosen to describe the population and characteristics of the intervention necessary for inclusion in the review. An example strategy for database searching is as follows:

1. (“older adult*” OR “elder*” OR “older person*” OR “older people*” OR “dementia”) AND
2. (“story” OR “stories” OR “storytelling” OR “biographi*” OR “biography*”) AND
3. (“digital” OR “multimedia” OR “virtual”)

A preliminary search of the databases mentioned above has been conducted to ensure that the search strategy is viable and the scope of the search is feasible. An example preliminary search of MEDLINE and EMBASE via Scopus produced 170 records based on the following query string:

(TITLE-ABS-KEY (“older adult*” OR “elder*” OR “older person*” OR “older people*” OR “dementia”) AND TITLE-ABS-KEY (“story” OR “stories” OR “storytelling” OR “biographi*” OR “biography*”) AND TITLE-ABS-KEY (“digital” OR “multimedia” OR “virtual”))

Finally, we will examine the reference lists of all included studies to identify any relevant studies that may have been missed from the original search.

### Data Collection and Analysis

#### Selection of Studies

The titles and abstracts of studies produced by the combined database search will be collated using reference management software, and duplicates will be removed. The first author will screen the titles and abstracts to remove reports that are irrelevant, before retrieving full-text reports of the remaining entries. A coauthor acting as the second reviewer will independently screen at least 25% of the titles and abstracts, and disagreements will be resolved through discussion, consulting a third reviewer to reach consensus where needed. The first author will then screen the full-text reports against the eligibility criteria for inclusion in the review, noting reasons for study exclusion. Again, a coauthor acting as the second reviewer will independently screen at least 25% of the full-text reports against the eligibility criteria, and disagreements will be resolved through discussion, consulting a third reviewer to reach consensus where needed. If more information is required, we will contact study authors where possible. The study selection process will be documented using the PRISMA flow chart, reporting the number of studies resulting from each stage of selection and the reasons for study exclusion [28].

#### Data Extraction and Management

We will use a pilot-tested standardized data collection form to extract and manage data from the included studies. Extracted data will include publication information (eg, name of authors, title, and country of origin), study design (eg, quantitative such as randomized and nonrandomized trials, pilot trials, open trials, and case studies; qualitative; and mixed methods), participant information (eg, sample size, age, gender, health conditions, and source of participants such as community or care settings), health-related outcomes for quantitative studies (eg, reported descriptive statistics and significance levels), health-related outcomes for qualitative studies (eg, reported theme-level findings), process (eg, length of participation and level of involvement in production) and product (eg, length of story and content of story) characteristics, and reported implementational challenges to digital storytelling. If there are multiple reports of a single study, such reports will be identified and the extracted data will be collated and presented as findings from a single study.

One reviewer will independently extract study data from all included studies. A second reviewer will independently extract data from at least 25% of the included studies. If there are discrepancies, these will be resolved by discussion, and a third reviewer will be consulted to reach consensus where necessary. Where possible, we will contact the study authors if more information is needed.

#### Data Synthesis

Given the anticipated heterogeneity in study designs, populations, and outcomes, a meta-analysis will not be feasible. As such, a narrative synthesis approach of the aggregate data will be used [29]. We will use tables to summarize and present participant demographics, health-related outcomes, and process and product characteristics.

Furthermore, we will describe the participant information, process characteristics, product characteristics, and factors related to the implementation that are shared among studies and are unique to individual studies or specific health-related outcomes.

#### Assessment of the Methodological Quality and Risk of Bias of the Included Studies

To assess the methodological quality of the included studies, the Mixed Methods Appraisal Tool (MMAT) will be used [30]. The MMAT is designed to concurrently assess the quality of studies with different methods, including qualitative, quantitative, and mixed methods studies. A detailed presentation of the ratings on each criterion, rather than presenting overall numerical scores, will be carried out to provide a more sensitive evaluation of the methodological quality. To increase validity, a coauthor acting as the second reviewer will assess the quality of at least 25% of the included studies. Disagreements will be resolved through discussion and consensus, consulting a third reviewer where necessary.

## Results

Systematic searches, data extraction and analysis, and writing of the systematic review are expected to be completed by the end of 2019.

## Discussion

Globally, the number of older adults is rapidly rising [1]. Challenges faced by the aging population today contribute to their poor psychological well-being, and many older adults live with several psychological comorbidities [2-4]. Innovative interventions such as digital storytelling are increasingly being utilized, and there is a need to evaluate the evidence base for such interventions.

Applying the methods outlined in this protocol, the aim of the proposed systematic review is to examine the use of digital storytelling with older adults. Primarily, the study aims to examine documented health-related outcomes for older adults. It also aims to present a summary of the various methods for digital storytelling and digital story products that emerge from the interventions. The systematic review will also assess the methodological quality of the evidence presented and will provide a discussion of the limitations of the review. Importantly, the findings from this review aim to assist in developing a model for digital storytelling interventions that is tailorable to older adults for specific health-related outcomes and has the capacity to be effective and implementable. The findings of this study will be published in a peer-reviewed journal and potentially presented at academic conferences.

## Abbreviations

MMAT: Mixed Methods Appraisal Tool
PRISMA: Preferred Reporting Items for Systematic Reviews and Meta-Analyses
